# Supplementary material for: Comparative multiomics analysis of cell physiological state after culture in a basket bioreactor
Source: Sci Rep. 2022 Nov 23;12:20161. doi: 10.1038/s41598-022-24687-4 (PMC9686226; doi:10.1038/s41598-022-24687-4)

_023666038.1:2783503:2784255:+@NW_023666038.1:2783503:2785847:+@NW_023666038.1:2785728:278584

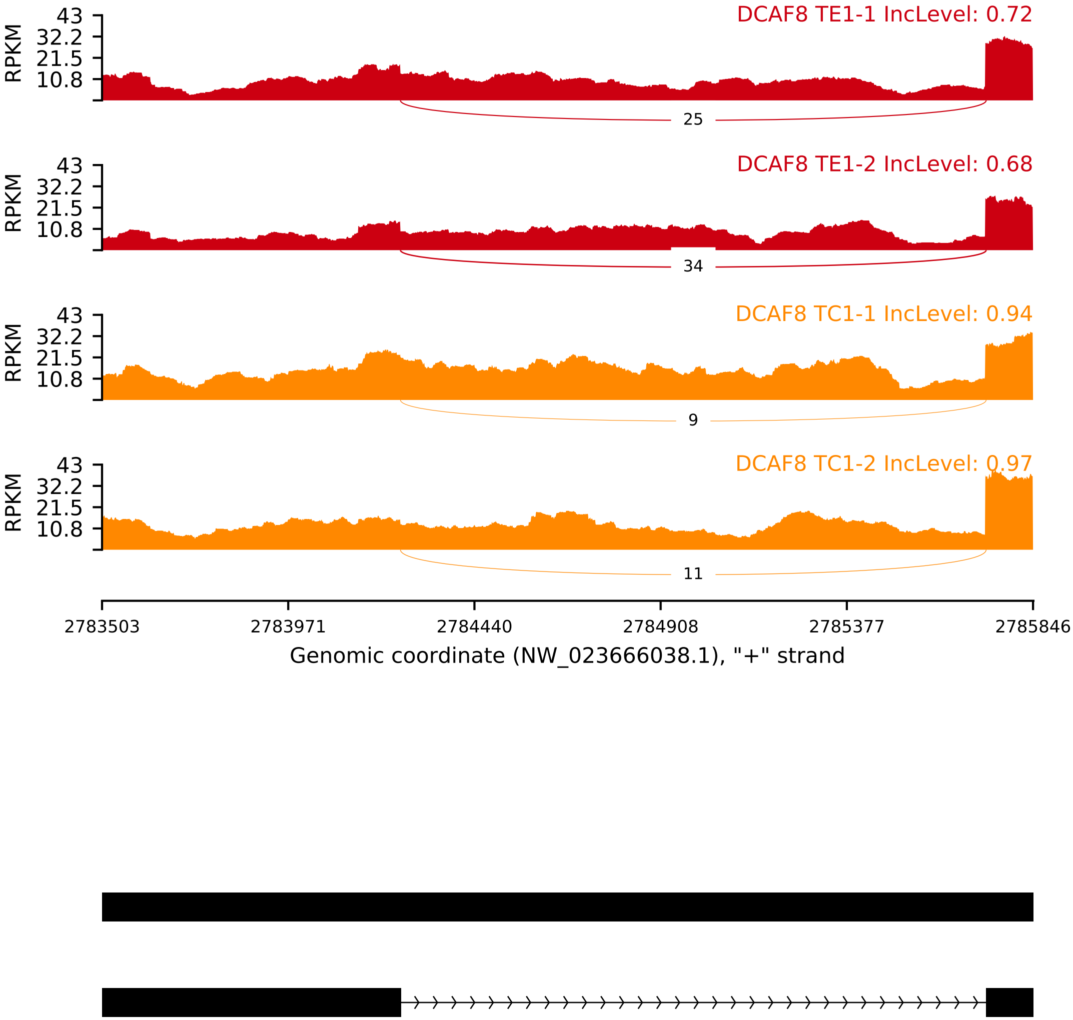

Supplement: Supplementary file 1 — Supplementary Information 1. [file 41598_2022_24687_MOESM1_ESM.zip › raw data/Transcriptomic raw data/7.AS/2.dasplot/TE1vsTC1/RI/6_DCAF8_NW_023666038.1_2783503_2784255_+@NW_023666038.1_2783503_2785847_+@NW_023666038.1_2785728_2785847_+.pdf]
